# A Hospital Appeal

**Published:** 1887-02-12

**Authors:** 


### A Hospital Appeal.

The following "Appeals" are selected from a number of replies sent to us by those competing for the Quarterly Prizes. We commend them to the consideration of those who are able and willing to give a practical response.

Christ's image hangs upon the wall,
His patient hands are outward spread,
As if their blessing fain would fall
On every waiting, empty bed.
The sun looks in with kindly ray—
The soft breeze comes and goes again—
It seems to say, "I fain would stay,
To soothe the weary in their pain."
O learn from Him on yonder tree,
Who left His sick to me and thee!—A. M. E.

May those who have of wealth abundant store,
Remember in their need the helpless sick and poor ;
Of this world's goods they can take none away,
But while on earth their bounty may display.
Aid Hospitals like Guy's, St. George's, London,
And gain at last a crown ; "Thy deeds are well done."

Patient.

How often in the midst of toil,
Or when engaged in play,
Some sudden ill may come and spoil
Perhaps for many a day,
In young or old, the strongest life,
That seemed so safe from pain ;
And yet the doctor's drug or knife
Leads it to health again.
Who, then, will help the Hospital,
Of funds so much in need ?
Relieve the sick – list to the call,
Kind friend, while now they plead.—H. J. C.

"None liveth to himself alone." Were this more fully realised, what a different state of things we should see around us, more especially with regard to our poorer brethren ! We should anxiously care for them ; and to support Hospitals—those noble embodiments of Christian charity—would be regarded not only as a duty, but a privilege ; remembering that the poor are especially dear to our Lord, and what is done for them He counts as done for Himself.—Tabs.

Help for the Hospital, I pray you to give,
To prove how the helpless we wish to relieve ;
Fainting and sick they come to that door
That should ever stand open to succour the poor.
But wards must be closed and nurses dismiss'd,
Unless those who have health and wealth will assist.
With faces so pale, weak and weary their step,
Shall we send them away without hope, without help ?

Paddy.

To all kind sympathising hearts I now appeal ;
To those who dearly prize their country's weal ;
A General Hospital's a common good,
It helps all men as one great brotherhood.
It knows no difference of creed or race,
Suffering is all that's needed to secure a place.
It would be sad so good a work should fail
For want of funds—Let charity prevail.—K. M.

Hark ! ye who are running the race for gold,
Spare me a moment, a tale I'll unfold ;
The wards they are shut for the want of wealth,
To give to many the blessings of health.
Fathers, mothers (breadwinners) are ill,
The little ones, too, need the doctor's skill.
Let your heart speak, I am sure 'tis not cold,
It whispers,—just listen !—"Give of your gold."
Your kind sympathy, too, is needful to all,
So spread far and wide the Hospital call.—Gretta.

